# PPARgamma: A Potential Intrinsic and Extrinsic Molecular Target for Breast Cancer Therapy

**DOI:** 10.3390/biomedicines9050543

**Published:** 2021-05-13

**Authors:** Giuseppina Augimeri, Daniela Bonofiglio

**Affiliations:** Department of Pharmacy, Health and Nutritional Sciences, University of Calabria, 87036 Cosenza, Italy; giusy.augimeri@gmail.com

**Keywords:** peroxisome proliferator-activated receptor gamma, PPARγ ligands, breast cancer, breast tumor microenvironment, thiazolidinediones, polyunsaturated fatty acids

## Abstract

Over the last decades, the breast tumor microenvironment (TME) has been increasingly recognized as a key player in tumor development and progression and as a promising prognostic and therapeutic target for breast cancer patients. The breast TME, representing a complex network of cellular signaling—deriving from different stromal cell types as well as extracellular matrix components, extracellular vesicles, and soluble growth factors—establishes a crosstalk with cancer cells sustaining tumor progression. A significant emphasis derives from the tumor surrounding inflammation responsible for the failure of the immune system to effectively restrain breast cancer growth. Thus, effective therapeutic strategies require a deeper understanding of the interplay between tumor and stroma, aimed at targeting both the intrinsic neoplastic cells and the extrinsic surrounding stroma. In this scenario, peroxisome proliferator-activated receptor (PPAR) γ, primarily known as a metabolic regulator, emerged as a potential target for breast cancer treatment since it functions in breast cancer cells and several components of the breast TME. In particular, the activation of PPARγ by natural and synthetic ligands inhibits breast cancer cell growth, motility, and invasiveness. Moreover, activated PPARγ may educate altered stromal cells, counteracting the pro-inflammatory milieu that drive breast cancer progression. Interestingly, using Kaplan–Meier survival curves, PPARγ also emerges as a prognostically favorable factor in breast cancer patients. In this perspective, we briefly discuss the mechanisms by which PPARγ is implicated in tumor biology as well as in the complex regulatory networks within the breast TME. This may help to profile approaches that provide a simultaneous inhibition of epithelial cells and TME components, offering a more efficient way to treat breast cancer.

## 1. Introduction

Emerging studies indicate that breast cancer is a complex disease, in which dynamic molecular exchanges between intrinsic neoplastic cells and the extrinsic surrounding stroma sustain tumor progression. Developing new therapeutic agents targeting both components of the cancer may provide the means to potentially achieve robust and durable outcomes. The work of our research group was devoted to dissecting the molecular mechanisms involved in the initiation and progression of endocrine-related cancers, with the main goal of identifying novel markers and potential therapeutic targets for these diseases. Here, we highlight the role of peroxisome proliferator activator receptor gamma (PPAR) γ as an avenue to be explored to provide an outlook for the future of breast cancer research.

## 2. PPARγ as a Molecular Target for Breast Cancer Therapy

PPARγ, belonging to the nuclear receptor sub-family of PPARs, is a transcription factor encoded by the *PPARG* gene on chromosome 3p25.2 in humans. The protein is broadly expressed with relatively high levels in the adipose tissue but also in various epithelial cell types, as well as in numerous cells of the immune system. PPARγ is structurally organized in five different domains: the N-terminus domain (A/B domain), which includes the ligand-independent activation factor 1 (AF-1) region; the central DNA-binding domain (C domain) responsible for the binding of PPARγ/agonist to the PPAR response elements (PPRE) within promoter and enhancer regions of its response genes; the D domain, which connects the C domain to the ligand-binding domain (E domain); and the F domain, localized in the C-terminus, which contains the ligand-dependent activation domain (AF2), involved in the docking of coactivator proteins in response to ligand stimulation [1,2,3]. In the absence of ligands, PPARγ, which is localized into the cytoplasm, interacts with a complex of co-repressor proteins, such as the silencing mediator of retinoic acid and the thyroid hormone receptor (SMRT), and the nuclear receptor corepressor complexes (N-CoR) preventing PPARγ activation [4,5]. The activation of PPARγ occurs by natural or synthetic ligands, such as omega (ω)-3 polyunsaturated fatty acids (PUFAs) and thiazolidinediones (TZDs), respectively. Upon binding to its agonists, PPARγ forms a heterodimer with the retinoid X receptor (RXR) enabling the translocation into the nucleus to bind the PPRE in the PPARγ target genes. Transcriptional control of such genes depends on multiprotein coregulatory complexes recruited to the PPREs, including PPARγ coactivator 1-α (PGC-1α) and binding protein p300 (EP300), both of which remodel the chromatin structure and enable the binding of the RNA polymerase to the promoter region, allowing the initiation of the genetic transcription [6]. Ligand activation of PPARγ regulates the transcription of genes that are involved in the control of energy and glucose homeostasis, as well as lipid metabolism and adipocyte differentiation [7,8,9]. Indeed, TZD compounds are hypoglycemic agents used as second-line medication in the treatment of type 2 diabetes mellitus due to their ability to ameliorate insulin resistance and lower circulating levels of insulin, reducing the risk of tumor development through the inhibition of insulin-like growth factor (IGF) signaling [10].

Over the last decades, PPARγ has also received considerable attention for its involvement in breast cancer tumorigenesis, even though its controversial role has been described. In specific types of cancers, including bladder and colon carcinoma, the activation of PPARγ resulted in genomic alterations that promoted tumorigenesis. However, the molecular mechanisms underlining these effects still remain unknown [11,12,13]. Interestingly, growing evidence suggests that PPARγ functions as a tumor suppressor in several tumors, including breast carcinoma [14,15,16,17]. In estrogen receptor (ER)- and progesterone receptor (PR)-positive cells, ER/PR-negative and human epidermal growth factor receptor (HER2)-positive cells, and triple-negative breast cancer cells, it has been reported that ligand-activated PPARγ modulates the activation of tumorigenic cascades, leading to inhibition of cell growth, migration, invasiveness, and metastatic properties [18,19,20,21,22]. Understanding these context-specific, multi-dimensional interactions can lead to a sophisticated, mechanism-based, rational approach to designing molecular cancer therapeutics. For instance, we identified the molecular mechanism by which PPARγ activated by ligands such as ω-3 PUFAs and their derivatives and thiazolidinediones participates in the regulation of vast gene expression networks. PPARγ does this by modulating effectors of the cell cycle, intrinsic and extrinsic apoptosis, and the autophagic process inducing breast cancer cell death and reducing cell growth [19,23,24,25,26,27]. PPARγ agonists may also act as negative regulators of cancer growth by the interaction with the IGF system and its down-stream pathways, such as mitogen-activated protein kinase (MAPK), phosphoinositide 3-kinase (PI3K), and the mechanistic target of rapamycin (mTOR) pathways [10]. Moreover, in Erα-positive breast cancer cells, PPARγ activation, via the upregulation of PTEN, shuts down the signals of the PI3K/Akt signaling cascade, mechanistically inducing growth arrest; PPARγ-mediated transcriptional events linked to cell cycle regulation can repress cyclin D1 expression [28] and upregulate p53 protein expression and its effector p21 in breast cancer cells [24]. Moreover, substantial evidence demonstrates that in hormone-dependent breast cancer cells, ERα binding to PPRE elements represses PPARγ transactivation, implying that a functional crosstalk between the two receptors affects breast cancer progression [18]. Conversely, PPARγ ligands enhance the efficacy of antitumoral drugs in ERα-positive breast cancer cells [29], highlighting the clinical value of these compounds in the adjuvant therapeutic strategies for breast cancer patients. However, besides inducing cell cycle arrest, PPARγ ligands promote breast cancer cell death through the activation of two pathways of apoptosis. One feature of activated PPARγ is the ability to trigger apoptosis through mitochondrial dysfunction and reactive oxygen species (ROS) production [30]. Moreover, by stimulating the release of the cytochrome *c* from the mitochondria into the cytoplasm and the cleavage of caspase 3,7 and poly (ADP-ribose) polymerase (PARP), PPARγ ligands can also activate the intrinsic apoptotic pathway. In addition, ligand-activated PPARγ binding to the Sp1 sequence located within the FASL gene promoter upregulates FASL expression, leading to caspase 8 cleavage, apoptotic cell death, and the extrinsic apoptotic process via a direct involvement of the FAS/FAS ligand (FASL) signaling pathway [19]. Intriguingly, our research group has demonstrated that natural PPARγ ligands, such as ω-3 PUFAs conjugates, trigger autophagy through a sequela of events consisting of: (1) the dissociation of the Beclin-1/Bcl2 complex, leading to an increase in Beclin-1 protein; (2) the reduction of the phosphorylation of p38; and (3) the enhancement of the microtubule-associated protein light chain 3 (LC-3) levels, as a specific membrane marker for the detection of early autophagosome formation [31]. However, autophagy represents only the first early event of PPARγ-dependent cell death since prolonged treatments with the ω-3 PUFAs and their conjugates induce intrinsic apoptosis through the cleavage of caspase 9 [21] and the upregulation of the tumor suppressor molecule syndecan-1 (SDC-1) in breast cancer cells [32].

A powerful advantage of the therapeutic use of PPARγ ligands is that these molecules could be potentially included in multidrug approaches. Indeed, PPARγ agonists exert a synergic effect with drugs, such as the selective cyclooxygenase-2 (COX-2) inhibitors, in inducing growth inhibition and apoptosis [33]. In addition, the combination of both PPARγ and RXR agonists induces growth inhibition and apoptosis in breast cancer cells [34]. We also reported that PPARγ and RXR agonists at low concentrations induce the intrinsic apoptotic pathway via p53 transcriptional activity in breast cancer cells. Indeed, we demonstrated that the PPARγ ligand rosiglitazone and the RXR ligand 9-*cis*-retinoic acid transactivate the tumor suppressor p53 promoter gene and enhance p53 protein expression and its target gene p21, increasing the release of the cytochrome c from the mitochondria into the cytoplasm and the cleavage of caspase-9, thus inducing the intrinsic apoptotic pathway [25]. Another mechanism of action by which both RXR and PPARγ agonists can also activate the intrinsic apoptosis is independent of p53 transcriptional activity and occurs through the formation of a p53–Bid complex at the mitochondria promoting apoptosis [35].

One of the biggest challenges for activated PPARγ-based therapeutic strategies is that these molecules have strong efficacy in inducing autophagy through the upregulation of the hypoxia-inducible factor 1 (HIF1α) [36], in triggering apoptosis [21], and in inhibiting the migration and invasion process by upregulating the expression of E-cadherin [22] in triple-negative breast cancer cells. This indicates that PPARγ agonists could be a good therapeutic tool for the management of the more aggressive breast cancer subtypes.

We now understand that PPARγ is expressed and functions not only in epithelial breast cancer cells but also in other components of the breast TME, including cancer-associated fibroblasts (CAFs) and tumor-associated macrophages (TAMs) interfering with breast tumor progression [20,37,38]. Specifically, activated PPARγ decreases the expression of C-X-C chemokine receptor type 4 (CXCR4) in CAFs, which represent the principal source of stromal cell-derived factor 1 alpha (SDF-1α) production, inhibiting their migratory capabilities and interfering with the autocrine and paracrine signaling loop acting to sustain breast tumor progression [20]. On the other hand, natural PPARγ agonists have been proven to re-educate TAMs, reducing cytokine production and attenuating inflammation and the pro-tumorigenic milieu in the breast TME [38].

The activated PPARγ receptor has been also found to counteract cell migration and invasion by regulating the secretion of soluble factors and the chemokine/receptor networks in the TME. In particular, the matrix metalloproteinases-9 (MMP9), a protease implicated in the extracellular matrix degradation, and CXCR4 were downregulated in breast cancer cells [20,39]. In both cases, the molecular mechanisms have been identified in the regulation of their promoter genes, highlighting that PPARγ functions as a gene modulator and determinant of tumor cell fate. However, as recently reviewed by us, the role of PPARγ as a tumor suppressor in the breast cancer microenvironment needs to be fully understood for further investigations [23,40].

Based on in vitro findings, PPARγ ligands were tested in murine breast cancer xenograft models where they proved to be effective in counteracting tumor growth, without inducing toxic effects either in combined PPARγ/RXR treatments or alone compared to controls [34,41,42]. Interestingly, we also highlighted the in vivo ability of activated PPARγ to antagonize the breast tumor growth induced by leptin in nude mice [26]. Regarding the natural PPARγ ligands, dietary supplementation with ω-3 PUFAs reduced tumor burden in rats in which mammary carcinogenesis was induced, showing the concomitant increase in PPARγ protein expression [43].

The promising findings obtained from the experimental in vitro and in vivo models provided the rationale for the evaluation of PPARγ agonist-based therapeutics in breast cancer clinical trials. A phase II study of a PPARγ agonist TZD, troglitazone, was carried out in metastatic breast cancer patients who displayed few clinical benefits, as the advanced stage of disease resulted in a breast cancer refractory to cytotoxic therapy [44]. Since troglitazone was withdrawn due to the high risk of hepatotoxicity, it was superseded by other TZD drugs, such as pioglitazone and rosiglitazone [23]. In a pilot study performed in early-stage breast cancer patients, the short-term administration of rosiglitazone failed to reduce breast tumor proliferation, reduced insulin resistance, and increased serum adiponectin levels, which are considered breast cancer risk factors [45]. It is worth pointing out that in this study, a decreased nuclear PPARγ expression was detected in the breast tumor tissue of patients treated with rosiglitazone, suggesting the rosiglitazone-mediated effects on breast cancer cell signaling [45]. Despite the lack of effectiveness of clinical evidence, we speculate that longer term and/or a combined therapy with other anti-cancer compounds may improve anti-tumoral effects of synthetic PPARγ agonists in breast cancer patients. In contrast, dietary ω-3 PUFAs appeared to have beneficial effects in the prevention, treatment, and control of the side effects of chemotherapy in breast cancer patients who participated in several clinical trials that have been completed or are still ongoing (www.clinicaltrials.gov, accessed on 27 April 2021). To date, 40 clinical studies are registered in the ClinicalTrials.gov databases when the search is restricted to “breast cancer” and “omega-3”.

Interestingly, from Kaplan-Meier analysis (www.kmplot.com, accessed on 15 April 2021)), it emerged that high PPARγ expression indicates better prognosis in breast cancer patients followed for 10 years. Specifically, high *PPARγ* mRNA expression was found to be significantly correlated with longer median overall survival (OS) (HR = 0.69 (0.57–0.84), *p* = 0.00022), recurrence free survival (RSF) (HR = 0.82 (0.74–0.91), *p* = 0.00014), post-progression survival (PPS) (HR = 0.79 (0.63–1), *p* = 0.05), and distant metastasis free survival (DMFS) (HR = 0.78 (0.67–0.91), *p* = 0.0017) for all breast cancer patients.

However, the prognostic significance of PPARγ in breast cancer is clearly different depending on its subcellular localization. Recent data have shown that nuclear expression of PPARγ is associated with longer OS, whereas detection in the cytoplasm is a prognostically unfavorable factor in disease-free and OS in various carcinoma, including in breast cancer [46,47]. These results indicate that the functional form of PPARγ may exert a potential protective role in breast cancer patients.

**Figure 1:**
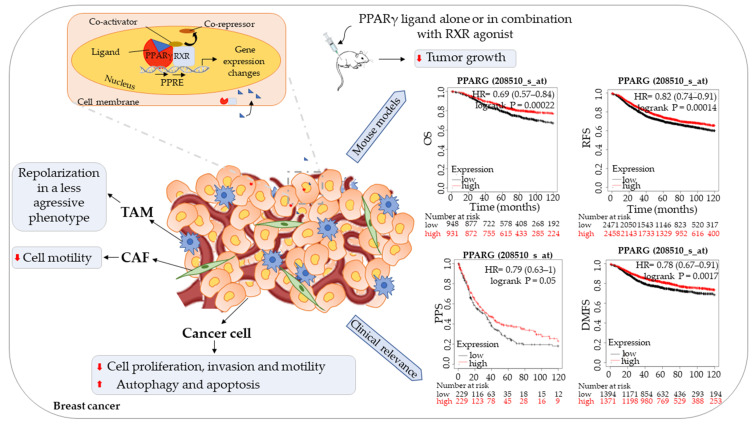
Schematic illustration showing the role of PPARγ in breast cancer. Ligand-activated PPARγ induces gene expression changes, affecting breast cancer cells, tumor-associated macrophage (TAM), and cancer-associated fibroblast (CAF) behavior in in vitro experiments. Administration of PPARγ ligands alone or in combination with retinoid X receptor (RXR) agonists reduces tumor growth in in vivo models. PPARγ expression in clinical samples of breast cancer is positively associated with overall survival (OS), recurrence-free survival (RFS), post-progression survival (PPS), and distant metastasis-free survival (DMFS) (www.kmplot.com, accessed on 15 April 2021), representing a potentially favorable prognostic marker.

Taking advantage of all these findings and the Kaplan–Meier survival curves summarized in Figure 1, studies using synthetic ligands or natural molecules that are devoid of side effects and that modulate PPARγ signaling should be planned for the management of breast cancer.

## 3. Conclusions

Despite significant knowledge on the role of PPARγ as a breast tumor suppressor in basic science, translation of these findings into human clinical trials has provided few encouraging results and needs to be further investigated. The major causes of failure of the synthetic PPARγ ligands for breast cancer treatment in clinical studies are the lack of effectiveness and the poor safety profiles that were not predicted in preclinical and animal studies. As synthetic PPARγ agonists showed several adverse effects, clinical use of natural PPARγ compounds may be a safe alternative for breast cancer patients. In addition, evaluation of the expression level of PPARγ and its subcellular localization in early-stage breast tumors should be considered a tailored selection criteria for patients to develop more effective translational and precision medicine strategies for breast cancer patients.
